# Improving consultations for persistent musculoskeletal low back pain in orthopaedic spine settings: an intervention development

**DOI:** 10.1186/s12891-021-04783-8

**Published:** 2021-10-21

**Authors:** Kathrin Braeuninger-Weimer, Naffis Anjarwalla, Alison McGregor, Lisa Roberts, Philip Sell, Tamar Pincus

**Affiliations:** 1 Department of Psychology, Royal Holloway, University of London, Egham, Surrey UK; 2 Department of Orthopaedics, Wexham Park Hospital, Slough, Berkshire UK; 3 Department of Surgery and Cancer, Imperial College London, London, UK; 4 School of Health Sciences, University of Southampton & University Hospital Southampton NHS Foundation Trust, Southampton, Hampshire UK; 5 Department of Orthopaedics, Leicester University Hospitals, Leicester, UK

**Keywords:** Musculoskeletal low back pain, Spinal care, Reassurance

## Abstract

**Background:**

There is a need to improve consultations between patients with persistent musculoskeletal low back pain and orthopaedic spine clinicians when surgery is not indicated. Poor communication and lack of education about self- management in these consultations have been shown to be associated with increased distress and higher subsequent health care seeking.

**Aim:**

To develop a standardised intervention to improve spine care consultations for patients for whom surgery is not beneficial.

**Method:**

The intervention was developed in six stages. The first three stages included: interviews with patients, an interactive workshop with clinicians from a mix of disciplines, and interviews with spine clinicians about their perspective of the recommendations, their perceived difficulties and potential improvements. Information from these stages was synthesised by an expert panel, creating a draft intervention structure and content. The main features of the intervention and the materials developed were then reviewed by patients and spine clinicians. Finally, the research team incorporated the recommended amendments to produce the intervention.

**Results:**

In total, 36 patients and 79 clinicians contributed to the development of the intervention. The final intervention includes three components: a pre-consultation letter with information suggesting that surgery is one possible intervention amongst many, introducing the staff, and alerting patients to bring with them a potted history of interventions tried previously. The intervention includes short online training sessions to improve clinicians’ communication skills, during the consultation, in reference to listening skills, validation of patients’ pain, and use of appropriate language. Clinicians are also supplied with a list of evidence-based sources for advice and further information to share with patients. Finally, post consultation, a follow up letter includes a short summary of the patients’ clinical journey, the results of their examination and tests, and a reminder of recommendations for self-management.

**Conclusion:**

The intervention includes aspects around patient education and enhanced clinician skills. It was developed with input from a multitude of stakeholders and is based on patients’ perceptions of what they would find reassuring and empowering when surgery is excluded. The intervention has the potential to improve the patients care journey and might lead to changes in practice in spine clinicians.

## Introduction

Persistent musculoskeletal low back pain (PMLBP) remains the leading cause of disability globally [1] and an urgent global public health concern [2]. Clinical practice guidelines reduced the emphasis on the use of routine imaging, pharmacological care/medication, and surgery [3, 4], instead key national guidelines promote the use of a biopsychosocial framework to inform assessment and management, including combined physical and psychological programmes and advice and support for self-management in relation to behavioural, psychological and social factors [5]. Buchbinder and colleagues [6] call for action in moving away from the emphasis of the biomedical approach, and argue that PMLBP is partly iatrogenic and the exposure to health care, especially western biomedical approaches to examination and management of low back pain, can sometimes have harmful consequences in relation to beliefs and reducing resilience to disability, as indicated by studies with Indigenous [7] and assimilated populations in high-income countries [8].

The biomedical model has limitations for this persistent disabling condition. Patients are often subjected to expensive imaging, which frequently does not improve outcomes but may lead to further unnecessary treatment that may be detrimental [9]. For those patients who fail to respond to first line conservative interventions (advice, anti-inflammatory medication, exercise and manual therapy), care is sometimes escalated to more invasive, expensive and potentially harmful interventions that hold limited evidence despite carrying substantial risks [10], such as opioids [11], injections [12], and surgery [13]. However, because surgery is often viewed as a last stop for people with PMLBP, and because spinal surgeons are often viewed as medical authorities by patients, these settings also present an opportunity for education and change. While many see the focus of the challenge in stopping the use of harmful and wasteful practices [6], this must be done while ensuring access to effective and affordable health care [14]. This means that when spinal clinicians, such as spinal surgeons and advanced practice practitioners (APP’s), explain to patients that surgery is not indicated, they need to offer acceptable alternatives, and ensure that patients feel reassured, rather than dismissed [15]. This can present a challenge, as the majority of spinal surgeons are not trained to treat patients who do not require surgery [16], despite patients viewing them as the final authority for back pain [15, 17]. Although reassurance is recommended by guidelines for PMLBP [18], it is a neglected area of research with limited advice on how to reassure patients [19]. There is emerging evidence [15, 17, 20] about the need to improve consultations between people with PMLBP and orthopaedic spine clinicians when surgery is excluded, to ensure that reassurance is effective and alternatives are discussed. Current practice does not offer optimal care to patients: recent evidence [15, 20] indicate that poor communication and lack of education about self- management in these consultations are associated with increased distress and higher subsequent health care seeking.

This study aimed to develop an intervention to improve consultations in which surgery was not indicated, by synthesising evidence, patients’ experience and perceived need, and clinicians’ perceptions of their own difficulties and of changes that would enable them to offer better care.

## Methods

### Design and recruitment

**Fig. 1:**
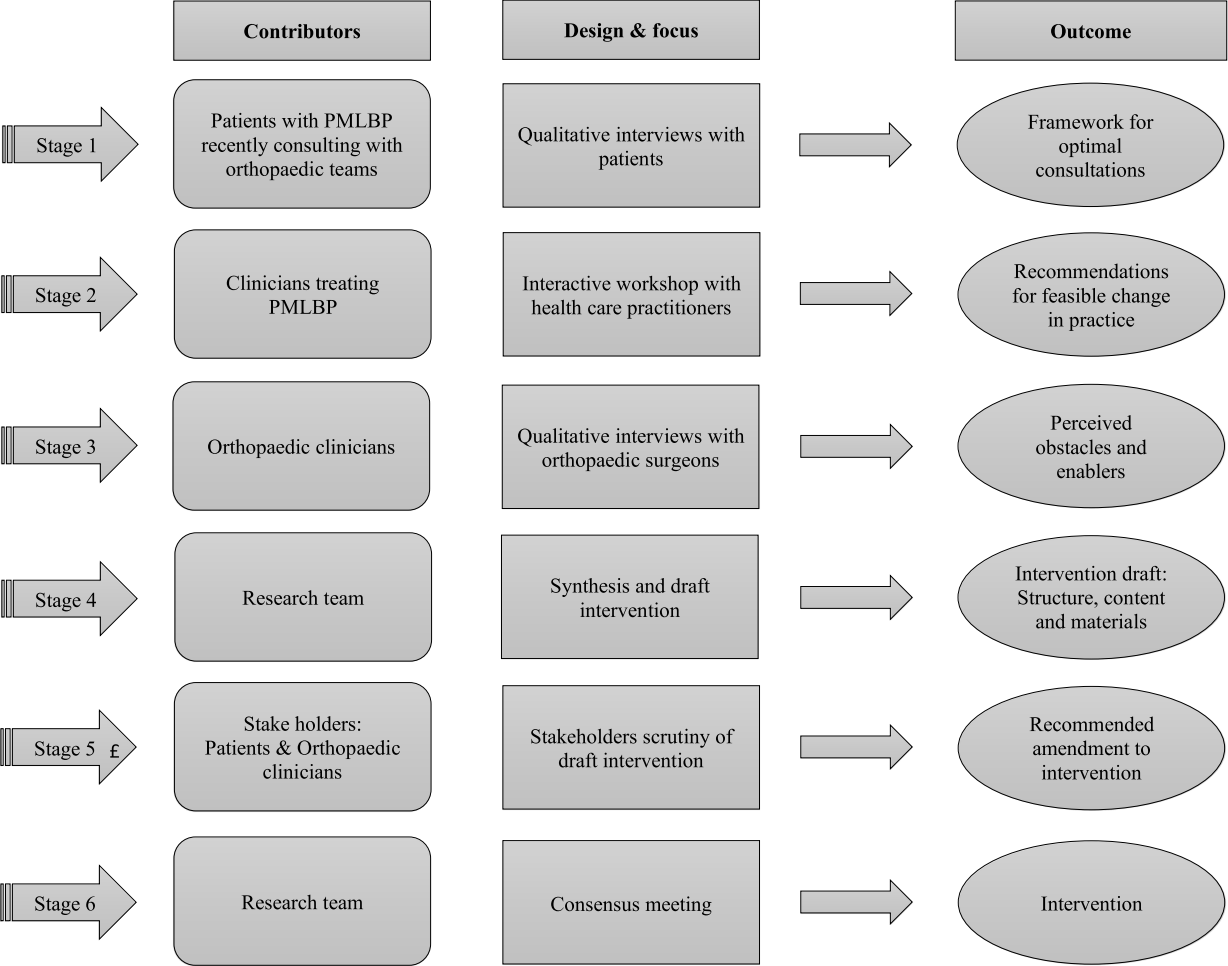
Intervention development stages

We considered the relevance and importance of each reporting item outlined by a guide for developing complex health interventions at the start and throughout the development process [21]. Following this guide, our development process was undertaken in parallel, dynamic, interactive, creative, and open to change format by revisiting components regularly as the intervention evolved. The design of this intervention development consensus study involved six stages (see Fig. 1). In order to reach an acceptable reflection of common practice in secondary care, a mixture of clinicians of various age groups and with different work experience was used (e.g. spinal surgeons, APP’s**,** registrars and fellows). All the clinicians in this study practice in the NHS in the United Kingdom (UK). The study was granted ethical approval from NHS Bloomsbury Research Ethics Committee (20/LO/0290) and the ethics committee at Royal Holloway, University of London.

#### Stage 1: qualitative interviews with patients

The starting point was constructed in interviews with patients (*n* = 30) who had recently consulted in outpatient clinics for PMLBP, in which surgery was excluded. This stage of the study has been published in detail elsewhere [15]. The aim of this stage was to elicit from patients their perception of an optimal reassuring consultation. The interviews focused on patients’ perceptions of their consultations, and their impact on their physical and mental well-being. Interviews were analysed using grounded theory and framework analysis. The findings suggested that patients wanted clinicians to provide effective reassurance to enable them to feel sufficiently empowered to self-manage their pain. Specifically, the behaviours they emphasised most frequently were grouped into four domains: showing comprehensive data gathering specific to patients, including their clinical history, life style, and current physical status; showing concern and empathy, while also demonstrating that they were experienced and understood the problem; avoidance of empty reassurance statements about positive prognosis while diminishing the problem, but instead offering validation of pain and suffering; and finally offering cognitive reassurance, through explanations and a management plan that was clear and personal.

#### Stage 2: interactive workshop with health care practitioners

This was followed by an interactive workshop in the context of a national conference (Annual Scientific Meeting (ASM) British Pain Society on 1st May 2019). Participants were 36 back pain clinicians from mixed disciplines. The aim of the workshop was to explore the feasibility and delivery method of each item produced by the patients in stage 1 in the context of practice in the UK National Health Service. The frame work consists of four domains of clinicians’ behaviours: adequate and comprehensive data gathering; good communications including empathic messages and listening skills; avoiding generic optimistic messages that are perceived as dismissal of suffering, and instead, providing validation of pain; and a clear explanation with an informative plan for future management. This framework combined with an overview of the previous literature was presented at a workshop. The workshop was facilitated by three of the authors in this paper (TP, NA, KBW). All clinicians who attended a parallel workshop participated in the definition of the consensus workshop material. Following the presentation of previous evidence, clinicians were randomly allocated to one of four groups. Each group was allocated to discuss one of the four domains in the ‘reassurance framework’. Participants were asked, for each item within their domain, to reach a consensus about the viability and optimal way of delivering change in practice. The recommendations were then discussed with the entire workshop group (see Table 1). The recommendations were then categorised by two of the research team (KW and TP) into changes in the health-care system, versus changes in the consultation, which were both considered to bring about positive change.

**Table 1:** Interactive workshop findings

Changes in service	Need for training
1. Encouraging hospitals transition to digital wold and the creation of summary e-notes that are easily assessable and quick to read. 2. Sending out clear written pre-appointment letters, stating reason for consultation and professional title to manage patients’ expectations. 3. Referral and post-consultation letters should be written to the patient and copied to the HCPs. 4. Letters should be written in an easy language. 5. Surgeons should not be the person of contact for patient who are not offered surgery. 6. Joint-triaging with the whole team to ensure affective triaging to the right practitioner. 7. Employing APP’s for the discharging communication as they have more time and are better suited. 8. Involving other health care professionals, such as nurses in the care of patients, to convey that surgery is not indicated but that other treatments may be suitable. 9. Making clear that surgeons only provide surgical opinion. 10. Need for consistent messages across different pathways or creating a single pathway. 11. Joint clinics – clinics with multidisciplinary staff running parallel so patients can smoothly be transitioned from one to another. 12. Revision of clinic schedules to allow more time for those patients. 13. Allowing follow-up appointments to update patients on potential new evidence-based interventions.	1. Clinical reasoning to why examination may not be needed. 2. Time-efficient reassurance techniques. 3. Breaking of ‘bad news’ to patients in the face of uncertainty. 4. Reflective, supportive, validating, and motivational communication skills (empathy, building rapport, reflective listening, validation, how to ‘presume less and ask more’, and how to convey support messages like ‘I can’t help you, but the next step is you going here…’). 5. Conveying medical information in layman’s terms in written or spoken form.

#### Stage 3: qualitative interviews with spine surgeons

Stage three consisted of qualitative interviews with 24 spinal clinicians, including 16 surgeons, 3 registrars/fellows, and 5 APP’s working under NHS secondary care settings [17], aiming to explore their experience of consultations in which surgery was excluded, and their perceptions of the patients’ framework and the recommendations produced in the interactive workshop. Participants were recruited through a snowball sampling method and all worked in an orthopaedic outpatient setting as part of the National Health Service (NHS). The interview content was divided into two parts. The first part is published in full elsewhere, and describes participant’s perceptions of their difficulties in communicating with patients when surgery is not an option [17]. In the second part of the interview, clinicians were presented with the findings from stage 1–2 of the intervention development and asked to discuss each item, in reference to whether they felt that the items make sense, would be useful, and feasible to implement. They were also asked how well the framework is currently managed, what might improve it, and what they perceive to be common obstacles for optimal delivery. The transcribed interviews indicated two meta themes: Difficulties, and Enablers. Surgeons described challenges around the choice of terminology and labels for PMLBP, and explaining test findings. They also described difficulties working with psychologically vulnerable patients, and managing expectations in reference to cure seeking through surgery. They felt that these difficulties could be reduced through management of expectations at earlier stages of the care journey, and through provision of training to improve communication skills and understanding of psychological issues. They also suggested changes to the care pathway, including use of routine imaging, triaging, and access to direct referral elsewhere, including other non-surgical practitioners in the team.

#### Stage 4: synthesis and draft intervention

The research team, consisting of two psychologists, two physiotherapists, and two spine orthopaedic surgeons, produced a synthesis of the findings from stages 1–3. The aim was to produce a draft of the structure, content and materials of the intervention. Two aspects emerged from the data: intervention components targeting patients (pre and post consultation information) and those targeting clinicians (training needs). The initial letter templates were developed by one of the authors (LR), who has extensive research experience in this field. The structure from a standard hospital appointment invitation letter was used as a template for the pre-consultation letter. Stage 1–3 informed on the changes that were made to the standard letter, which included instructions on preparation of a description of the clinical journey to date, what to expect of the consultation, and a section on who the patient might see and why. For the post-consultation letter, a standard discharge and referral letter was used as template. In addition, the surgeons in the team (NA, PS) informed on what GP’s might expect to see from a discharge letter. This information in combination with stage 1–3 informed on the additional content, which included personalised summaries of the impact of pain, a summary of previous management options, explanation of why surgery is not offered, explicitly stating a diagnosis where possible, a summary of the agreed management plan and a list of online resources to aid patients in their self-management process.

#### Stage 5: stakeholders scrutiny of draft intervention

The draft intervention was reviewed in one-to-one virtual interviews by stakeholders, including patients (*n* = 6) and clinicians (orthopaedic surgeons *n* = 11, neurosurgeon *n* = 1, APP *n* = 1). Patients and public individuals (PPI): all six participants were patients who had previously consulted an orthopaedic clinician for their back pain and were discharged without further treatment. Participants were presented with the pre- and post-consultation draft letters and asked what they thought of each section, which aspects they felt work and whether it would ease their care journey in secondary care, what is still missing, and where is the potential for this to go wrong. Patients recommended some changes to wording describing back pain. Clinicians were recruited in a snowball opportunity sampling. Participants were presented with the pre- & post- consultation letter and shown a short psychoeducational video outlining the importance of validation, as an example of the training material. They were asked to provide feedback on the content, detail, format, usefulness, risk and acceptability of the intervention. They were asked which aspects work, what is still missing, what are the chances of this helping, and where is the potential for it to go wrong? Their comments were collated, using both verbatim quotes and numbers for consensus. Recommended changes were all structural, and focused on the order and style in which information was presented.

#### Stage 6: consensus meeting

The final stage consisted of a consensus meeting with the research team, who discussed and integrated all comments and suggestions from the stakeholders to finalise the intervention framework to guide the delivery. Each meeting began with the presentation of an overview of the feedback comments, followed by a detailed discussion over each item that reflected divergence. Detailed discussions were held on items until the expert panel reached consensus to implement changes. This led to changes in the pre- and post- consultation letters as well as the training material.

## Results

In total, 36 patients and 79 clinicians contributed to the intervention development. Those involved described the material as useful, clear, and likely to be well accepted by patients and clinicians. The developed intervention has three broad objectives: to clarify and ease patients’ care journey through managing their expectations, to improve clinicians’ communication and effective reassurance methods during consultations, and to provide patients with clear and useful information that will support their self-management. These objectives were addressed in three time points: pre-consultation, in the appointment letter; during consultation, through training provided to clinicians; and post consultation, in a follow up letter. The final intervention framework, including the objectives, methods, and outcomes, are provided in Table 2.

**Table 2:** Intervention content

Time	Objectives	Method	Short term patient outcomes
**Pre-consultation: patient letter**	• To manage patients’ expectations & ease their communication with clinicians during the consultation through preparation	• Providing information on who is the consultation team, who they might get to see, and why • Making clear that surgery is not necessarily indicated • Requesting patients to bring a list of previous treatments & questions	• Realistic expectations • Reduced anxiety • Greater buy-in to alternative to surgery • Reduced disappointment when surgery is not offered
**During- consultation: short educational online video**	• To improve the delivery of clear and concise information • To increase HCP confidence in delivering the message that surgery is not recommended, by providing information on other options tailored to individuals • To increase clinicians’ skills in delivering effective reassurance, including validation of patients’ pain experience	• Education about appropriate use of terminology • Information about eliciting and responding to psychological issues • Education describing timely and effective validation • Advice on provision of other interventions and options that might help people manage their pain	• Increased perceived reassurance • Reduced anxiety and intention to further seek care • Compliance and buy-in to self-management • Knowledge about alternative options
**Post-consultation: patient letter**	• To increase patients’ understanding & accurate recall of what was discussed in the consultation • To increase options and support for pain management by providing resources	• Personalised summary of the impact of pain on patients’ life • Summary, in lay terms, of the investigation results • Summary of previous interventions and management attempts • Summary of discussion, recap of patients’ goals • Re-cap of options for self-management and other support to living with pain	• Comprehension and acceptance of consultation decisions • Satisfaction with information provided • Reduced intensions to re-consult

### Pre-consultation letter

The aim of the pre-consultation letter is to prepare patients realistically, manage their expectations, and ease communication for clinicians.

In addition to information that is typically present in standard hospital appointment letters, the letter includes instructions on preparation of the clinical history of back pain treatment, which entailed asking patients to bring a list of treatment attempts in addition to the list of medication that they are on. This assists clinicians in avoiding recommendations of treatments and actions that have already been tried. The letter also entails a line reminding patients to bear in mind when deciding what to wear (e.g. underwear) since they may be asked to remove their clothing for the physical examination. This was a remark made mainly from female patients, but clinicians also felt it was important to remind patients before coming to their appointments. A section explaining that surgery is not necessarily indicated for back pain was included as well as information on who they should expect to see at the consultation. This section highlights the different team members in the team, e.g. spinal surgeons, neurosurgeon, registrars, fellows, or APP’s, who might be extended scope physiotherapist or nurses, and that the patient may be seen by any of these team members. Of note, the letter does not specify which of these professionals will be seen. The end of the letter comprises of a lined space left empty for patients to note down questions in relation to their condition or its management that they feel was important for them to ask the clinician. This section was important for patients, because they reported that they often felt walking away from these consultations that they wished they had asked specific questions. Likewise, clinicians’ feedback also indicated agreement over this part of the letter because they felt it might help structure the communication during consultations, especially for nervous patients who may also feel further rushed as a result of lack of time during busy clinic schedules.

### During the consultation – training material

The aim of providing clinicians with a single short educational online video is to evoke changes in practice through improving the delivery of clear and concise information, with appropriate terminology that is easily understood. It also aims to increase clinician’s confidence in delivering the message that surgery is not often recommended, by providing information on other options tailored to the individual and how to use imaging effectively. Thus, it is a tool set to increase clinicians’ skills in delivering effective reassurance, including validation of patient’s pain experience.

Clinicians felt the training should be introduced by briefly outlining the difficulties of effectively reassuring patients with troublesome PMLBP, with a focus on the evidence that reassurance is associated with patient’ outcomes.

Current practice involves showing patients their MRI scan as a justification to why surgery is not indicated. The training enhances practitioner’s skills by providing:

- education about what language should and should not be used.
- a short, acceptable and understandable biopsychosocial explanation of pain and its management.
- examples of ways to elicit and respond to patients’ concerns and psychological issues.
- an explanation of validation, with examples of how to deliver it effectively.

As part this training practitioners are provided with a list of evidence-based patient centred online resources, to use during the consultation or direct patients towards.

When asked to comment on the reassurance framework, orthopaedic clinicians, in stage 3 of the intervention development, cohesively agreed with the majority of the content and identified it as something they were already doing in their practice. However, ‘reading patients case notes’, ‘showing empathy’, ‘follow-up with letter’, and ‘offering open follow-up appointments’ were not considered as always feasible. Particularly, ‘offering follow-up appointments’ was considered as contradictory to one of the aims of the intervention, which entailed encouraging patients towards self-management.

Orthopaedic clinicians suggested it is important that some of the video material should be delivered by an experienced orthopaedic surgeon.

### Post-consultation letter

The post-consultation letter is a combination of sections pre-developed by the research team, and a section inserted by the consulting practitioners, based on individual information and communication during the consultation. The follow-up letter aims to increase patients understanding and accurate recall of what was discussed in the consultation. This should result in increased satisfaction with the information provided and reduce intentions to re-consult. It should provide written resources to aid self-management. Stakeholder patients noted that it may also serve as long awaited proof of their persistent disabling condition, which they can show to friends, family, and work colleges.

The letter begins by stating that it serves as a summary of the discussion during their consultation about the best way to treat patients’ pain. It summarises the impact of pain on the patient’s life, and lists the treatments that patients have tried so far.

Subsequently, the letter includes a paragraph summarising what the assessments and investigations showed and a simple explanation of the pain. To be noted here is that the phrase ‘your back is strong and stable’ should be avoided, since patients felt it is condescending and clinicians said it contradicts their constant advice for patients to stay active and do exercises to strengthen their backs. The letter explains that there are no serious signs of disease (cancer, infection, tumours, etc.), which patients often find reassuring. The letter initially stated that their diagnosis is known as ‘chronic low back pain’, however, patients felt it does not sound serious enough and evokes guilt in them for not going to work. Instead, they suggested to change the name to ‘persistent musculoskeletal low back pain’, which they felt sounded more serious and by indicating that it is a permanent condition, may reduce subsequent care and cure seeking. The letter states that surgery is not recommended, and suggests further non-surgical treatments as the most effective way forward. A summary of the discussion of the best way to improve symptoms is inserted, which also serves to remind patients to have realistic management goals. Finally, patients are provided with reliable resources that contain good information and support for their condition (e.g. websites, books, exercise videos (e.g. NHS app), back pain online communities, etc.).

## Discussion

This study developed a bespoke, standardised, brief and accessible intervention, through iterative research and discussion with the stakeholders, and with direction from both patients and clinicians trying to manage persistent musculoskeletal low back pain. The final intervention includes online training for clinicians in orthopaedic teams, and pre- and post- consultation letter templates. It provides a framework to the care journey and management of patients with PMLBP consulting for spinal surgery in orthopaedic settings when surgery is ruled out.

The intervention components were created through an iterative discussion with stakeholders, but they chime with published research on patient expectations, challenges in communication, validation, and difficulties around finding the optimal terminology to reassure patients.

### Patient expectations

Our stakeholders suggested that assessing and managing patients’ expectations needed to be addressed in the consultation [15]. The impact of patients’ expectation of treatment on outcomes is well documented [22]. Schrooten and Linton [23] have argued that changing patients’ expectations remains challenging, and that research should focus on improving education. Disconfirmation and modification of chronic pain patients’ expectations by communication with health care professionals is specifically outlined as a priority [23].

Communication as an integral part of the health care process often starts before the patient sets foot into the service and thus the initial approach to patients is crucial as it sets the tone that provides patients with a first impression of the services [24]. The idea of intervening through a pre-consultation letter has been tested with musculoskeletal outpatients in preparation for physiotherapy [24], but to our knowledge, has not been developed or tested for patients attending orthopaedic teams for PMLBP.

### Improving detection and discussions around psychological issues

There is evidence to suggest that spine specialists struggle at identifying psychosocial risk factors in patients with PMLBP [25] and orthopaedic surgeons are less likely to formally screen and refer patients for psychological treatments [26]. There is also a large body of evidence suggesting that communication skills, in general, could be improved in orthopaedic surgeons [27–34]. Within our study, spinal clinicians recognised that they do not have formal training or effective skills in assessing, screening, and discussing psychosocial factors with their patients, especially for those where surgery is not indicated, and thus identified it as a training need [17].

### Avoiding jargon and selecting appropriate language

There is evidence suggesting that orthopaedic surgeons often use jargon of language and terms that patients may find alarming [18, 34–36]. This use of language may also contribute to patient’s lack of understanding around test results, increase catastrophic beliefs, and result in further health care seeking. A study in Australia has developed a standardised method of interpreting imaging results in order to reassure musculoskeletal back pain patients [37], which can facilitate how imaging results are conveyed by clinicians in secondary care.

### The importance of empathic validation

The importance of empathic communication skills in consultation with PMLBP patients has been supported by the literature [38]. In particular, orthopaedic surgeons have been characterised as low and infrequent empathic clinicians [30, 39], with a decline in empathy originating during the clinical years of medical school [40]. A qualitative study exploring physiotherapists perception of empathy during musculoskeletal clinical encounters proposed a model of acquiring, developing, and delivering empathy within the constraints of clinical settings [41]. There is evidence to suggest that educational interventions, including communication skills training, may increase the level of empathy among medical students [42]. Likewise, training empathetic validation to medical students results in improved communication with patients living with pain, which suggests that such training for other health care providers may also be feasible [43]. Furthermore, the importance of validation in the management of patients with persistent disabling back pain has been supported [15, 44–46]. Validation is associated with improved pain-interference and depression [47], reduced negative effect and frustration/ anger [48], higher accurate recall [49] and proposed to sooth negative affect and increase disclosure, which may promote problem solving and shared decision making [45].

### Providing and enhancing buy-in to self-management plans

Self-management for low back pain is often described as a model of care where patients use tools to manage and monitor their own condition [50, 51]. Patients often autonomously apply self-care strategies that include exercise and self-medication, yet ideally clinicians provide evidence-based information about different management strategies that patients may test in the effort to find what works best for them for managing their condition on a daily basis [52]. The post-consultation letter includes individualised options, and agreed decisions about gaol setting in terms of daily routines. Goal setting is considered imperative to effective self-management because it entails measuring progress towards achieving a functional outcome that is meaningful to patients life [53]. The findings from a Cochrane systematic review of 39 RCTs involving 2846 patients mainly with persistent musculoskeletal disorders and chronic pain, indicates that goal setting may have positive effects for psychosocial outcomes (e.g. improved health related quality of life, emotional status, and self-efficacy), rather than physical ones [54].

### Strengths and limitations

The project was undertaken by a multidisciplinary research group and embedded in the development process was consideration of valuable patient’ and multisite clinician’ input at several stages. The intervention proposed is unique, evidence-based, and it includes aspects that both patients and practitioners consider, which will improve the experience of the consultation and empower patients to manage their pain better in the future. It was developed through a structured iteration process with stakeholders, but is also informed by theoretical frameworks [15]. However, we recognise that just because aspects of the intervention were desired, does not mean their implementation will be effective. Behavioural change in healthcare professionals remains a major challenge [55] and interventions targeting behavioural change in health care professionals are often unsuccessful [56]. Thus, it remains unclear whether orthopaedic clinicians will accept and engage with the training as intended. In addition, the intervention as designed includes three components, and targets both patients and practitioners. As such, and in common with many complex interventions, a randomised controlled trial of the intervention as a whole would not be able to establish which components of the intervention were effective. Finally, caution is required about generalising the intervention. This intervention was developed with patients and practitioners in the UK, who had all experience a specific health system (NHS). It is unlikely to generalise to other systems and settings, although some components in the intervention may be useful in other contexts. This study prepares the ground for a proof of concept and a randomised controlled trial to test the effectiveness of the intervention.

## Conclusion

The intervention has the potential to improve spine care. It includes providing patients with education and information about their condition and management, tailored to the individual. It also includes aspects that convey enhanced communication skills to spine clinicians, which may lead to changes in practice that improve consultations.

## Data Availability

All data generated during this study are included in this published article. The datasets (qualitative interviews, Stage 3) analysed during the current study are not publicly available as this would compromise confidentiality but are available from the corresponding author on reasonable request.

## Abbreviations

APP: Advanced practice practitioner
NHS: National Health Service
PMLBP: Persistent musculoskeletal low back pain
UK: United Kingdom

## References

[1] Wu A, March L, Zheng X, Huang J, Wang X, Zhao J (2020). Global low back pain prevalence and years lived with disability from 1990 to 2017: estimates from the Global Burden of Disease Study 2017. Ann Transl Med.

[2] Hartvigsen J, Hancock MJ, Kongsted A, Louw Q, Ferreira ML, Genevay S (2018). What low back pain is and why we need to pay attention. Lancet..

[3] National Institute for Health and Care Excellence (NICE) (2021). Chronic pain (primary and secondary) in over 16s: assessment of all chronic pain and management of chronic primary pain. NG193.

[4] Qaseem A, Wilt TJ, McLean RM, Forciea MA (2017). Noninvasive treatments for acute, subacute, and chronic low back pain: a clinical practice guideline from the American College of Physicians. Ann Intern Med.

[5] Foster NE, Anema JR, Cherkin D, Chou R, Cohen SP, Gross DP (2018). Prevention and treatment of low back pain: evidence, challenges, and promising directions. Lancet..

[6] Buchbinder R, van Tulder M, Öberg B, Costa LM, Woolf A, Schoene M (2018). Low back pain: a call for action. Lancet..

[7] Lin IB, O’Sullivan PB, Coffin JA, Mak DB, Toussaint S, Straker LM (2013). Disabling chronic low back pain as an iatrogenic disorder: a qualitative study in Aboriginal Australians. BMJ..

[8] Bui Q, Doescher M, Takeuchi D, Taylor V (2011). Immigration, acculturation and chronic Back and neck problems among Latino-Americans. J Immigr Minor Health.

[9] Webster BS, Choi Y, Bauer AZ, Cifuentes M, Pransky G (2014). The cascade of medical services and associated longitudinal costs due to nonadherent magnetic resonance imaging for low back pain. Spine..

[10] Kent P, O’Sullivan P, Smith A, Haines T, Campbell A, McGregor AH (2019). RESTORE-cognitive functional therapy with or without movement sensor biofeedback versus usual care for chronic, disabling low back pain: study protocol for a randomised controlled trial. BMJ Open.

[11] Deyo RA, Von Korff M, Duhrkoop D (2015). Opioids for low back pain. BMJ..

[12] Staal JB, de Bie RA, de Vet HCW, Hildebrandt J, Nelemans P (2009). Injection therapy for subacute and chronic low Back pain. Spine..

[13] Fritzell P, Hägg O, Nordwall A (2003). Complications in lumbar fusion surgery for chronic low back pain: comparison of three surgical techniques used in a prospective randomized study. A report from the Swedish lumbar spine study group. Eur Spine J.

[14] Clark S, Horton R (2018). Low back pain: a major global challenge. Lancet..

[15] Braeuninger-Weimer K, Anjarwalla N, Pincus T. Discharged and dismissed: a qualitative study with back pain patients discharged without treatment from orthopaedic consultations. Eur J Pain. 2019:1464–74. 10.1002/ejp.1412. 10.1002/ejp.1412 31069890

[16] Dhillon KS (2016). Spinal fusion for chronic low Back pain: a ‘magic bullet’ or wishful thinking?. Malays Orthop J.

[17] Braeuninger-Weimer K, Anjarwalla N, McGregor A, Roberts L, Sell P, Pincus T (2021). Taking patients to the ice cream shop but telling them that they cannot have ice cream: a qualitative study of orthopaedic spine clinicians’ perceptions of persistent low back pain consultations. BMJ Open.

[18] NICE (2016). Low back pain and sciatica in over 16s: assessment and management.

[19] Linton SJ, McCracken LM, Vlaeyen JWS (2008). Reassurance: help or hinder in the treatment of pain. Pain..

[20] Braeuninger-Weimer K, Rooslien H, Anjarwalla N, Pincus T (2021). Reassurance and health care seeking in people with persistent musculoskeletal low back pain consulting orthopaedic spine practitioners: a prospective cohort study. Eur J Pain.

[21] O’Cathain A, Croot L, Duncan E, Rousseau N, Sworn K, Turner KM (2019). Guidance for reporting intervention development studies in health research (GUIDED): an evidence-based consensus study. BMJ Open.

[22] Peerdeman KJ, van Laarhoven AIM, Peters ML, Evers AWM (2016). An integrative review of the influence of expectancies on pain. Front Psychol.

[23] Schrooten MGS, Linton SJ (2017). Changing pain expectations: the role of social context and communication. Pain..

[24] Roberts L (2006). First impressions: an information leaflet for patients attending a musculoskeletal outpatient department. Physiotherapy..

[25] Patel MS, Lee KC, Dhake RP, Longworth S, Sell P (2021). Ability of Spine Specialists to Identify Psychosocial Risk Factors as Obstacles to Recovery in Patients with Low Back Pain-Related Disorders. Asian Spine J.

[26] Vranceanu AM, Beks RB, Guitton TG, Janssen SJ, Ring D (2017). How do Orthopaedic surgeons address psychological aspects of illness?. Arch bone Jt Surg.

[27] Frymoyer JW, Frymoyer NP (2002). Physician-patient communication: a lost art?. J Am Acad Orthop Surg.

[28] Kyle S, Shaw D (2014). Doctor-patient communication, patient knowledge and health literacy: how difficult can it all be?. Ann R Coll Surg Engl.

[29] Herndon JH, Pollick KJ (2002). Continuing concerns, new challenges, and next steps in physician-patient communication. J Bone Jt Surg.

[30] Levinson W, Chaumeton N (1999). Communication between surgeons and patients in routine office visits. Surgery..

[31] Levinson W, Roter DL, Mullooly JP, Dull VT, Frankel RM (1997). Physician-patient communication; the relationship with malpractice claims among primary care physicians and surgeons. J Am Med Assoc.

[32] Ambady N, LaPlante D, Nguyen T, Rosenthal R, Chaumeton N, Levinson W (2002). Surgeons’ tone of voice: a clue to malpractice history. Surgery..

[33] Levinson W, Hudak P, Tricco AC (2013). A systematic review of surgeon–patient communication: strengths and opportunities for improvement. Patient Educ Couns.

[34] Tongue JR, Epps HR, Forese L (2005). Communication skills for patient-centered care. Research-based, easily learned techniques for medical interviews that benefit Orthopaedic surgeons and their patients. J Bone Jt Surg..

[35] Kampa RJ, Pang J, Gleeson R (2006). Broken bones and fractures - an audit of patients’ perceptions. Ann R Coll Surg Engl.

[36] Bagley C, Hunter A, Bacarese-Hamilton I (2011). Patients’ misunderstanding of common orthopaedic terminology: the need for clarity. Ann R Coll Surg Engl.

[37] Karran EL, Yau YH, Hillier SL, Moseley GL (2018). The reassuring potential of spinal imaging results: development and testing of a brief, psycho-education intervention for patients attending secondary care. Eur Spine J.

[38] Cohen M, Quintner J, Buchanan D, Nielsen M, Guy L (2011). Stigmatization of patients with chronic pain: the extinction of empathy. Pain Med.

[39] Portalatín EL, Carrazana LF, Colon R, Abreu R, Rivera D, Lojo L (2018). Orthopaedic surgeon communication skills: perception of empathy and patient satisfaction through the use of anatomic models. J Am Acad Orthop Surg Glob Res Rev.

[40] Han JL, Pappas TN (2018). A review of empathy, its importance, and its teaching in surgical training. J Surg Educ.

[41] Allen MV, Roberts LC (2017). Perceived acquisition, development and delivery of empathy in musculoskeletal physiotherapy encounters. J Commun Healthc.

[42] Batt-Rawden SA, Chisolm MS, Anton B, Flickinger TE (2013). Teaching empathy to medical students. Acad Med.

[43] Linton SJ, Flink IK, Nilsson E, Edlund S (2017). Can training in empathetic validation improve medical students’ communication with patients suffering pain? A test of concept. Pain Rep.

[44] Linton SJ, Boersma K, Vangronsveld K, Fruzzetti A (2012). Painfully reassuring? The effects of validation on emotions and adherence in a pain test. Eur J Pain.

[45] Linton SJ (2015). Intricacies of good communication in the context of pain: does validation reinforce disclosure?. Pain..

[46] Edmond SN, Keefe FJ (2015). Validating pain communication: current state of the science. Pain..

[47] Edlund SM, Wurm M, Holländare F, Linton SJ, Fruzzetti AE, Tillfors M (2017). Pain patients’ experiences of validation and invalidation from physicians before and after multimodal pain rehabilitation: associations with pain, negative affectivity, and treatment outcome. Scand J Pain.

[48] Vangronsveld KL, Linton SJ (2012). The effect of validating and invalidating communication on satisfaction, pain and affect in nurses suffering from low back pain during a semi-structured interview. Eur J Pain.

[49] Carstens JKP, Boersma K, Schrooten MGS, Linton SJ (2017). Effects of validating communication on recall during a pain-task in healthy participants. Scand J Pain.

[50] Oliveira VC, Ferreira PH, Maher CG, Pinto RZ, Refshauge KM, Ferreira ML (2012). Effectiveness of self-management of low back pain: systematic review with meta-analysis. Arthritis Care Res.

[51] Carnes D, Homer KE, Miles CL, Pincus T, Underwood M, Rahman A (2012). Effective delivery styles and content for self-management interventions for chronic musculoskeletal pain: a systematic literature review. Clin J Pain.

[52] May S (2010). Self-management of chronic low back pain and osteoarthritis. Nat Rev Rheumatol.

[53] Mallick-Searle T, Sharma K, Toal P, Gutman A (2021). Pain and function in chronic musculoskeletal pain—treating the whole person. J Multidiscip Healthc.

[54] Levack WMM, Weatherall M, Hay-Smith EJC, Dean SG, McPherson K, Siegert RJ (2015). Goal setting and strategies to enhance goal pursuit for adults with acquired disability participating in rehabilitation. Cochrane Database Syst Rev.

[55] Grimshaw JM, Eccles MP, Lavis JN, Hill SJ, Squires JE (2012). Knowledge translation of research findings. Implement Sci.

[56] Johnson MJ, May CR (2015). Promoting professional behaviour change in healthcare: what interventions work, and why? A theory-led overview of systematic reviews. BMJ Open.

